# A Comparison of Doxorubicin-Resistant Colon Cancer LoVo and Leukemia HL60 Cells: Common Features, Different Underlying Mechanisms

**DOI:** 10.3390/cimb43010014

**Published:** 2021-05-22

**Authors:** Laura Locatelli, Alessandra Cazzaniga, Giorgia Fedele, Monica Zocchi, Roberta Scrimieri, Claudia Moscheni, Sara Castiglioni, Jeanette A. Maier

**Affiliations:** 1 Department of Biomedical and Clinical Sciences L. Sacco, Università di Milano, Via G.B. Grassi 74, 20157 Milano, Italy; laura.locatelli@unimi.it (L.L.); alessandra.cazzaniga@unimi.it (A.C.); giorgia.fedele@unimi.it (G.F.); monica.zocchi@unimi.it (M.Z.); roberta.scrimieri@unimi.it (R.S.); claudia.moscheni@unimi.it (C.M.); jeanette.maier@unimi.it (J.A.M.); 2 Interdisciplinary Centre for Nanostructured Materials and Interfaces (CIMaINa), Università di Milano, 20133 Milan, Italy

**Keywords:** doxorubicin, LoVo cells, HL60 cells, TRPM7, MagT1, ROS, mitochondria

## Abstract

Chemoresistance causes cancer relapse and metastasis, thus remaining the major obstacle to cancer therapy. While some light has been shed on the underlying mechanisms, it is clear that chemoresistance is a multifaceted problem strictly interconnected with the high heterogeneity of neoplastic cells. We utilized two different human cell lines, i.e., LoVo colon cancer and promyelocytic leukemia HL60 cells sensitive and resistant to doxorubicin (DXR), largely used as a chemotherapeutic and frequently leading to chemoresistance. LoVo and HL60 resistant cells accumulate less reactive oxygen species by differently modulating the levels of some pro- and antioxidant proteins. Moreover, the content of intracellular magnesium, known to contribute to protect cells from oxidative stress, is increased in DXR-resistant LoVo through the upregulation of MagT1 and in DXR-resistant HL60 because of the overexpression of TRPM7. In addition, while no major differences in mitochondrial mass are observed in resistant HL60 and LoVo cells, fragmented mitochondria due to increased fission and decreased fusion are detected only in resistant LoVo cells. We conclude that DXR-resistant cells evolve adaptive mechanisms to survive DXR cytotoxicity by activating different molecular pathways.

## 1. Introduction

Doxorubicin (DXR) is a widely used anthracycline-based antitumor agent for both solid and liquid tumors. However, the administration of DXR frequently results in the development of drug resistance, a critical hurdle in cancer treatment [1]. The mechanisms of chemoresistance are numerous and depend upon the acquisition of various properties by cancer cells. One of them is the expression of specific transporters that protect the neoplastic cells by pumping cytotoxic drugs out of the cells [2]. The best characterized drug efflux pump is P-glycoprotein (Pgp), a glycosylated 170 kDa transmembrane protein which belongs to the ATP-binding cassette family [3]. Physiologically, Pgp is expressed in the normal tissues among which the gut, kidney, placenta and brain [4], with the function of defending them from toxins and xenobiotics. Pgp overexpression in tumors is rather common and leads to reduced accumulation of widely used chemotherapeutics such as paclitaxel, vinca alkaloids and anthracyclines [5]. Clinical trials with Pgp inhibitors, however, did not yield successful results [6], partly because of toxicity and drug interaction [7] and partly because corollary mechanisms seem to contribute to cell resistance to antineoplastic drugs. One of these is the adaptive response of resistant cancer cells against oxidative stress, which is induced by several chemotherapeutics, including DXR [8], as a cytotoxic mechanism to eliminate neoplastic cells. In general, resistant cancer cells possess a richer antioxidant arsenal and, consequently, show lower amounts of reactive oxygen species (ROS) than sensitive cells [9]. We have previously shown that DXR-resistant LoVo colon cancer cells (LoVo-R) accumulate less ROS than their sensitive (LoVo-S) counterpart [10]. This might be due, in part, to a higher intracellular content of magnesium (Mg) [11], known to protect against oxidative stress [12]. Importantly, Mg is necessary to make ATP biologically active, and LoVo-R require remarkable amounts of ATP to extrude DXR through Pgp. We have also demonstrated that Mg homeostasis in LoVo-R is maintained through the overexpression of Magnesium Transporter protein 1 (MagT1) and the downregulation of Transient Receptor Potential Cation Channel Subfamily M Member 7 (TRPM7), two ubiquitously expressed Mg transporters [13]. Notably, the involvement of Mg transporters is well documented in several types of digestive cancers including colorectal adenocarcinomas [14]. In the cell Mg is stored primarily in the mitochondria [15], and, interestingly, we described different structural organization of these organelles in LoVo-R vs. LoVo-S [10].

The aim of our study was to compare two different models of DXR resistant cells, i.e., adherent vs. suspension tumor cells. To this purpose, we utilized colon carcinoma LoVo-R and -S and human promyelocytic leukemia HL60 cells sensitive or resistant to DXR (HL60-S and HL60-R) to identify possible common features that might turn into biomarkers of drug resistance, with consequent implications in diagnosis and therapy. 

## 2. Materials and Methods

### 2.1. Cell Culture

LoVo are APC/Ras mutant human colon carcinoma cells [16]. LoVo-S and LoVo-R (Istituto dei Tumori, Milano, Italy) [11,13] were cultured in DMEM containing 10% fetal bovine serum (FBS) and 2 mM glutamine at 37 °C and 5% CO_2_. HL60 are a promyelocytic cell line derived from human leukemia [17] bearing the amplification of c-myc [18] and the deletion of p53 [19]. HL60 were purchased by American Type Culture Collection (ATCC, Manassas, VA., USA). HL60-R were obtained by exposure to stepwise incremental concentrations of DXR as described [20]. Briefly, the cells were treated with concentrations of DXR ranging from 0.05 to 10 µg/ml DXR (Sigma Aldrich, St. Louis, MO, USA). The procedure was repeated until the cells survived a concentration of DXR as high as 10 µg/ml and required about 4 months. The cells were tested for DXR resistance every month by adding 10 µg/ml of DXR to the culture medium for 3 days (data not shown).

HL60-S and HL60-R were cultured in RPMI containing 10% FBS and 2 mM glutamine at 37 °C and 5% CO_2_. All the reagents used for cell culture were purchased from Euroclone (Milano, Italy). The cells were stained with trypan blue solution (0.4%) (Sigma Aldrich), and the viable cells were counted using a cell counter after different time points.

To obtain a transient downregulation of *TRPM7* in HL60-R, we used Lipofectamine RNAiMAX (Thermo Fisher Scientific, Waltham, MA, USA) according to the manufacturer’s recommendations in combination with the stealth siRNAs for *TRPM7* (FlexiTube GeneSolution GS54822 for *TRPM7*, Qiagen, Hilden, Germany). Non-silencing, scrambled sequences were used as controls. To inhibit TRPM7, HL60-R were exposed to 2-aminoethoxydiphenyl borate (2-APB, 12.5 μM, Thermo Fisher Scientific). To analyze DXR cytotoxicity after inhibiting TRPM7, HL60-R were counted using a cell counter as described above. 

All the experiments were performed three times in triplicate ± standard deviation (SD).

### 2.2. TUNEL Assay

The Click-iT TUNEL Alexa Fluor 488 Imaging Assay (Thermo Fisher Scientific) was performed on HL60 cells. The cells were seeded in 24-well plates and treated with 0–0.5–1 μg/ml of DXR. After 24 h, the cells were collected, counted and 100,000 cells for each sample were fixed in PFA 4% and then permeabilized in PBS containing 0.25% Triton X-100 (Sigma Aldrich) before performing the TUNEL reaction according to manufacturer’s instruction. At the end of the assay, the cells were incubated with Hoechst 33,342 (Thermo Fisher Scientific) for 10 min and then washed with PBS twice. The TUNEL Alexa Fluor 488 dye emission was monitored at 519 nm (λ_ex_ = 495 nm, λ_em_ = 519 nm) using Varioskan LUX Multimode Microplate Reader (Thermo Fisher Scientific) and then normalized on Hoechst emission (λ_ex_ = 350 nm, λ_em_ = 461 nm). The results are the mean ± SD of three independent experiments performed in triplicate. 

### 2.3. Western Blot Analysis 

Cells were lysed in lysis buffer (50 mM Tris-HCl pH 8.0, 150 mM NaCl, 1 mM EDTA, 1% Nonidet P40 Substitute). Protein concentration was determined using the Bradford reagent (Sigma Aldrich). Equal amounts of proteins were separated by SDS-PAGE and transferred to nitrocellulose membranes by using Trans-Blot Turbo^TM^ Transfer Pack (Bio-Rad, Hercules, CA, USA). Western blot analysis was performed using primary antibodies against Pgp, PON2, TXNIP, CYP D (Thermo Fisher Scientific), SOD2, OPA1 (BD Biosciences, St. Diego, CA, USA), TRPM7 (Bethyl, Montgomery, TX, USA), MagT1 (Abcam, Cambridge, UK), SIRT2 (Merck Millipore, Burlington, MA, USA), DRP1 (Cell Signalling, Danvers, MA, USA), β-actin and GAPDH (Santa-Cruz Biotechnology, Dallas, TX, USA). Secondary antibodies conjugated with horseradish peroxidase (Amersham Pharmacia Biotech Italia, Cologno Monzese, Italy) were used. The immunoreactive proteins were detected with Clarity^TM^ Western ECL substrate (Bio-Rad) and images were captured with a ChemiDoc MP Imaging System (Bio-Rad). Densitometry of the bands was performed with the software ImageJ (National Institute of Health, Bethesda, MD, USA). The Western blots shown are representative and the densitometric analysis was performed on three independent experiments ± SD.

### 2.4. Immunofluorescence and Confocal Imaging

LoVo were cultured on coverslips, while HL60 were cytospun on frosted microscope glasses (Thermo Fisher Scientific). The cells were then fixed in phosphate buffered saline containing 4% paraformaldehyde and 2% sucrose pH 7.6, permeabilized with Triton 0.3%, incubated with anti-CYP D immunopurified IgGs overnight at 4 °C, and stained with an Alexa Fluor 488 secondary antibody (Thermo Fisher Scientific). Rhodamine-conjugated phalloidin (Thermo Fisher Scientific) was used to visualize cytoskeleton and nuclei were stained with 4′,6-diamidino-2-phenylindole (DAPI) (Thermo Fisher Scientific). Finally, glass coverslips were mounted with moviol and images were acquired using a 63X objective in oil by a SP8 Leica confocal microscope (Leica Microsystems, Wetzlar, Germany).

### 2.5. Quantification of Total Intracellular Mg

After an overnight starvation in medium containing 2% FBS, total Mg content was assessed on sonicated cells by using the fluorescent chemosensor DCHQ5 as described [11]. Fluorescence intensities were acquired at 510 nm using the Varioskan LUX Multimode Microplate Reader (Thermo Fisher Scientific). Mg concentrations of the samples were obtained by the interpolation of their fluorescence with the standard curve performed using MgSO_4_. The experiments were performed three times in triplicate ± SD.

### 2.6. Reactive Oxygen Species Production

ROS production was quantified using 2′-7′-dichlorofluorescein diacetate (DCFH) (Thermo Fisher Scientific) [21]. The cells were exposed for 30 min to 20 µM DCFH solution and the emission at 529 nm of the DCFH dye was monitored using the Varioskan LUX Multimode Microplate Reader (Thermo Fisher Scientific). The results are the means of three independent experiments performed in triplicate ± SD. 

### 2.7. Statistical Analysis

Data are expressed as the mean ± SD. Western blots were performed at least three times. Fluorescence and TUNEL assays and cell counts were performed three times with three replicates in each experiment. The data were nonparametric and normally distributed and were analyzed using one-way ANOVA. The *p*-values deriving from multiple pairwise comparisons were corrected using the Bonferroni method. The statistical analysis was performed with the software GraphPad Prism. Statistical significance was defined as *p*-value < 0.05. Regarding the figures, * *p* < 0.05; ** *p* < 0.01; *** *p* < 0.001; **** *p* < 0.0001. 

## 3. Results

### 3.1. Characterization of HL60-R

**Figure 1:**
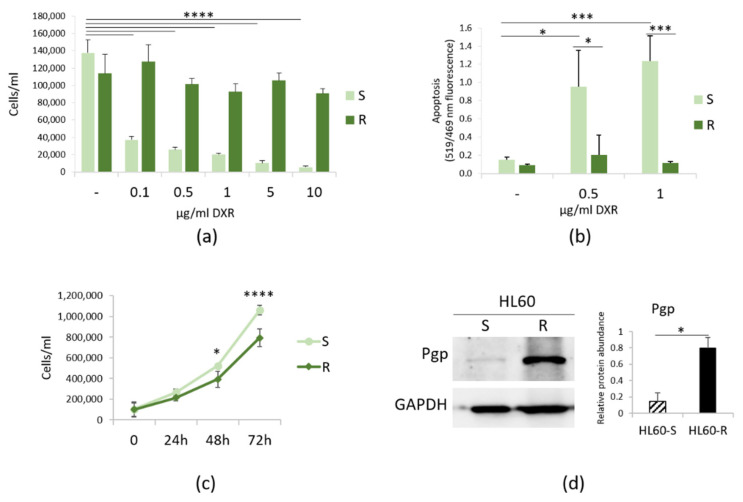
Characterization of HL60-R. (**a**) HL60-S and -R were cultured in the presence of different concentrations of DXR and counted after 24 h. (**b**) TUNEL assay was performed on HL60-S and -R treated for 24 h with DXR (0.5 and 1 μg/ml). (**c**) HL60-S and -R were cultured for 24, 48 and 72 h in their culture medium and counted every 24 h. (**d**) Western blot was performed on protein lysates using antibodies against Pgp. GAPDH was used as control of loading. A representative blot and densitometry obtained by ImageJ on three independent experiments ± SD are shown. * *p* < 0.05; *** *p* < 0.001; **** *p* < 0.0001.

Initially, we tested the behavior of HL60-S and -R cultured in the presence of various concentrations of DXR for 24 h. Figure 1a shows that HL60-S do not survive even at the lowest concentrations of DXR and, as demonstrated with TUNEL assay, they undergo apoptotic cell death (Figure 1b). Then, we compared HL60-R proliferation rate with HL60-S’s. The cells were cultured without DXR for various lengths of time. After staining with trypan blue, the viable cells were counted. As shown in Figure 1c, DXR resistance retards cell growth. Figure 1d shows that HL60-R upregulate Pgp as detected by Western blot. Even though HL60-R express MRP1 [22], we propose that such a massive increase of Pgp levels represents the principal resistance mechanism to DXR in HL60-R. 

### 3.2. ROS in Sensitive and Resistant LoVo and HL60 Cells

**Figure 2:**
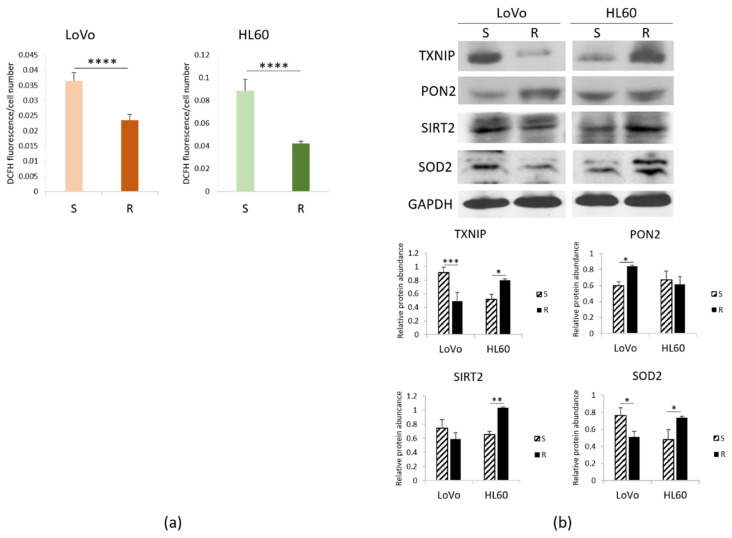
Redox balance in DXR sensitive and resistant cells. LoVo and HL60 were cultured for 24 h in their culture medium. (**a**) ROS production was measured by DCFH. Fluorescence at 529 nm was measured and normalized to the cell number. (**b**) Western blots were performed on protein lysates using antibodies against TXNIP, PON2, SIRT2 and SOD2. GAPDH was used as control of loading. A representative blot and densitometry obtained by ImageJ on three independent experiments ± SD are shown. * *p* < 0.05; ** *p* < 0.01; *** *p* < 0.001; **** *p* < 0.0001.

We measured ROS accumulation in resistant vs. sensitive cells and found it decreased in DXR-resistant LoVo and HL60 (Figure 2a). It is noteworthy that verapamil, which inhibits Pgp in vitro [23], did not exert any effect on DCFH accumulation.

To unveil the mechanisms involved, we investigated the levels of the pro-oxidant thioredoxin-interacting protein (TXNIP) and some anti-oxidant proteins, i.e., paraoxonase (PON) 2, NAD-dependent deacetylase sirtuin (SIRT) 2 and superoxide dismutase (SOD) 2. We detected lower amounts of TXNIP and SOD2 and higher levels of PON2 in LoVo-R than -S, as detected by Western blot. On the contrary, HL60-R upregulate TXNIP, SOD2 and SIRT2, while PON2 is not modulated (Figure 2b), thus suggesting that the upregulation of SOD2 and SIRT2 might counterbalance the pro-oxidant activity of TXNIP. These results suggest that resistant cells accumulate lower amounts of ROS through a different regulation of pro- and antioxidant proteins.

### 3.3. Mg Homeostasis in HL60-S and -R

**Figure 3:**
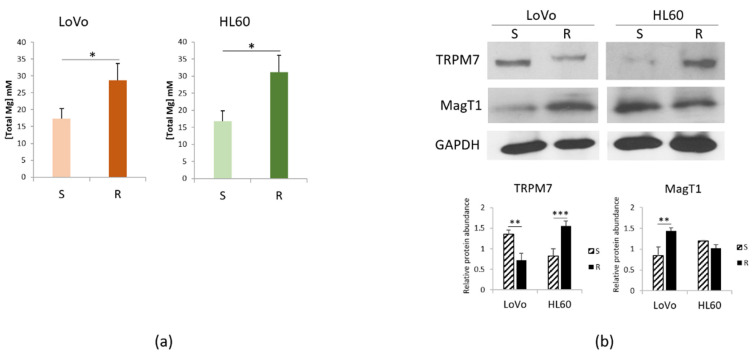
Mg homeostasis in DXR sensitive and resistant cells. LoVo and HL60 were cultured for 48 h in their culture medium. (**a**) Total intracellular Mg was measured. (**b**) Western blots were performed on protein lysates using antibodies against TRPM7 and MagT1. GAPDH was used as control of loading. A representative blot and densitometry obtained by ImageJ are shown. * *p* < 0.05; ** *p* < 0.01; *** *p* < 0.001.

Mg serves many fundamental functions within the cell among which protection against oxidative stress [12]. Consistently, we have previously reported that the concentration of total intracellular Mg is higher in LoVo-R than in LoVo-S [11]. Figure 3a shows that the same result is obtained in HL60, suggesting that a higher content of intracellular Mg might represent a signature of resistance to DXR.

Mg homeostasis is maintained through the intervention of Mg transporters. Figure 3b shows that LoVo-R downregulate TRPM7 and upregulate MagT1, thus confirming our previous report [13]. On the contrary, in HL60-R TRPM7 is markedly increased, whereas MagT1 is slightly but not significantly decreased.

**Figure 4:**
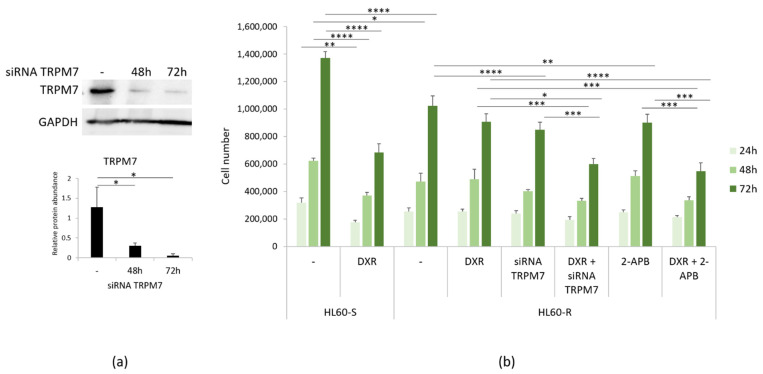
DXR cytotoxicity after inhibiting TRPM7. (**a**) HL60-R were transfected with a siRNA against *TRPM7* for 48 and 72 h or with scrambled sequences as control (-). Western blot using antibodies against TRPM7 was performed on cell extracts as described in the methods. GAPDH was used as control of loading. A representative blot and densitometry obtained by ImageJ ± SD are shown. (**b**) HL60-R were silenced or treated with 2-APB (12.5 μM) to inhibit TRPM7. The cells were then treated or not with DXR (10 µg/ml). HL60-S treated or not with DXR were used as controls. The cells were counted after 24, 48 and 72 h. The experiment was performed three times in triplicate ± SD. * *p* < 0.05; ** *p* < 0.01; *** *p* < 0.001; **** *p* < 0.0001.

We have previously shown that genetic and pharmacological inhibition of TRPM7 induces the acquisition of a DXR-resistant phenotype in LoVo-S [11]. Here we silenced *TRPM7* using specific siRNA in HL60-R. Western blot shows that TRPM7 was downregulated by siRNA (Figure 4a). We also exposed the cells to the TRPM7 channel blocker 2-APB (12.5 μM). HL60-R were counted after 24, 48 and 72 h. Figure 4b shows that the cells become more sensitive to the cytotoxic effects of DXR upon *TRPM7* silencing or chemical inhibition. These results indicate that, differently from LoVo [11], the downregulation/inhibition of TRPM7 increases HL60-R sensitivity to DXR.

### 3.4. Mitochondria in DXR-Sensitive or Resistant HL60 and LoVo

**Figure 5:**
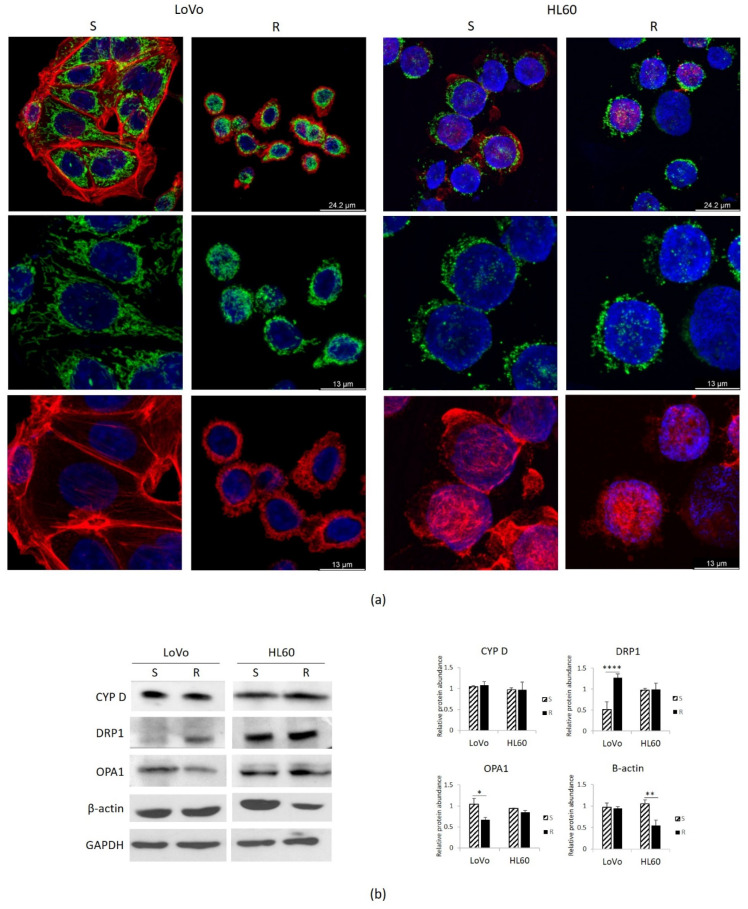
Mitochondria in DXR sensitive and resistant cells. LoVo and HL60 were cultured for 24 h in their culture medium. (**a**) LoVo were directly cultured on coverslip, while HL60 were cultured in flasks and then cytospun on glass coverslips. The cells were stained with CYP D to visualize the mitochondria (green), with rhodamine-conjugated phalloidin to visualize cytoskeleton (red) and DAPI to label the nuclei (blue). (**b**) Western blots were performed on protein lysates using antibodies against CYP D, DRP1, OPA1 and β-actin. GAPDH was used as control of loading. A representative blot and densitometry obtained by ImageJ are shown. * *p* < 0.05; ** *p* < 0.01; **** *p* < 0.0001.

Initially, we compared the morphology of cells and mitochondria in LoVo-R vs. -S [10] and in HL60-R vs. -S. The cells were stained with rhodamine-conjugated phalloidin and DAPI to visualize the actin filaments and the nuclei, respectively. As previously described, LoVo-R and -S are markedly different in cell size and morphology [10,13] (Figure 5a). LoVo-S are spindle shaped, with a central nucleus. LoVo-R are small and round, their nucleus is central. No significant differences in cell morphology were observed in HL60-R and -S (Figure 5a). The cytoskeleton is less abundant in HL60-R and this corresponds to lower amounts of total actin as shown by Western blot (Figure 5b). 

We then focused on mitochondria. In addition to producing ATP, mitochondria integrate signals from the environment, sense cellular stresses, regulate cell death signaling, store Mg, thus playing a crucial role in coordinating cell behavior [24,25]. We utilized antibodies anti cyclophilin D (CYP D), which is part of the permeability transition pore in the inner membrane of the mitochondria, to detect these organelles by confocal microscopy.

As previously described, LoVo-S exhibit a complex network of stretched mitochondria, while in LoVo-R the mitochondria are round (Figure 5a). No significant differences were observed in HL60-R and -S, which display round mitochondria diffusely distributed in the cytosol (Figure 5a).

Similar amounts of CYP D were detected in HL60-R and -S as well as in LoVo-R and -S by Western blot (Figure 5b), indicating that resistance does not impact on mitochondrial content. We then analyzed the amounts of Dynamin Related Protein-1 (DRP1), the crucial player of mitochondrial fission, and Optic Atrophy 1 (OPA1), which is required for the fusion of mitochondria. While no significant differences were detected between HL60-R and S, LoVo-R upregulate DRP1 and downregulate OPA1 (Figure 5b). These results suggest an increased fission and a reduced fusion in LoVo-R, and explain the differences in mitochondrial shapes between LoVo-R and -S.

## 4. Discussion

DXR is commonly used in the treatment of a variety of malignant neoplasms, including myelogenous leukemias [26] and colon cancer [27,28,29]. However, DXR can induce drug resistance with consequent poor patient prognosis and survival, partly through the upregulation of Pgp, which actively extrudes the drug out of the cell [30]. Nevertheless, Pgp inhibitors have minimal beneficial outcomes [31], thereby suggesting that other mechanisms are involved in the acquisition of the resistant phenotype. Obviously, understanding the molecular bases of chemoresistance is crucial to design efficacious therapeutic approaches. 

To get insights into this issue, we compared the behavior of human HL60 promyelocytic and colon cancer LoVo cells sensitive and resistant to DXR. 

Chemotherapeutics, including DXR, are designed to kill cancer cells by generating oxidative stress [32]. However, during chemotherapy, some cancer cells activate adaptive responses to protect themselves against oxidative species. As an example, MCF-7 and MDA-MB-231 cells upregulate pro-survival proteins upon exposure to DXR [33]. Moreover, in MDA-MB-231 DXR decreases ROS by increasing the amounts of SOD2 [34]. Accordingly, on the basis of a transcriptome analysis it was shown that DXR-resistant MCF-7 and MDA-MB-231 modulate 74 genes involved in oxidative stress [35]. Our results in LoVo-R and HL60-R are consistent with these findings. LoVo-R markedly downregulate TXNIP, an important redox regulator which is frequently reduced in cancer through epigenetic mechanisms [36]. Low amounts of TXNIP are protective for neoplastic cells. Indeed, TXNIP induces apoptosis by inhibiting the function of the thioredoxin system [37]. LoVo-R also downregulate SOD2, the principal scavenger of mitochondrial superoxide [38], while they upregulate PON2, a membrane-associated lactonase with antioxidant properties [39]. We propose that the different levels of these proteins result in lower amounts of ROS in LoVo-R than in LoVo-S. Similarly, HL60-R produce less ROS than HL60-S, although a different balance between pro- and antioxidant molecules is observed. In HL60-R, TXNIP, SOD2 and SIRT2 are upregulated. TXNIP was originally found as a target of vitamin D in HL60 cells and shown to inhibit the antioxidant activity of thioredoxin [40]. It is noteworthy that TXNIP overexpression significantly suppressed the proliferation of two acute myeloid leukemia cell lines, i.e., MOLM-13 and MV4-11 [41]. On these bases, we hypothesize that TXNIP upregulation might play a role in retarding HL60-R proliferation. SIRT2 is a member of the NAD+ -dependent class III histone deacetylase family and SIRT2 mediated-deacetylation is involved in maintaining redox homeostasis. It is of note that SIRT2 is expressed at higher levels in the relapsed patients with acute myelogenous leukemia than the newly diagnosed patients and, consistently, in HL60 downregulating Pgp [42]. Moreover, SIRT2 overexpression antagonizes the cytotoxicity of lapatinib in nasopharyngeal carcinoma [43]. We propose that high amounts of SOD2 and SIRT2 might counteract the pro-oxidant activity driven by the upregulation of TXNIP in HL60-R.

The role of Mg in cancer is complex and controversial [44] and very little is available about Mg and chemotherapeutic resistance. It is known that neoplastic cells accrue higher amounts of intracellular Mg and this seems to be even more accentuated in drug resistant cells, since both HL60-R and LoVo-R tend to accumulate more Mg than the corresponding sensitive cells. Since Mg is needed for the biological activity of ATP, it is feasible to explain the high content of Mg in resistant cells as the result of the high energetic requirements to extrude DXR. Intracellular Mg homeostasis is maintained through the activity of several channels and transporters [12]. We here confirm that LoVo-R downregulate TRPM7, a divalent cation channel fused to a C-terminal serine/threonine kinase, and upregulate MagT1, a Mg transporter also implicated in the post-translational transfer of glycans to proteins [13]. Consistently, MagT1 is overexpressed in colon cancer and seems to be associated with tumor metastasis and anticancer drug resistance [45]. On the contrary, HL60-R show higher amounts of TRPM7 than HL60-S, while MagT1 is not significantly modulated. While very little is known about MagT1 in cancer [45], much more is known about TRPM7. A single nucleotide polymorphism of TRPM7 (T1482I) has been linked to an increased breast cancer risk [46] and to the development of polyps that might progress to colon carcinoma [47]. Moreover, the overexpression of TRPM7 has been reported in glioblastoma and in prostatic, nasopharyngeal, pancreatic, breast and ovarian cancers [48]. Importantly, TRPM7 overexpression was linked with increased metastatic potential and poor clinical outcome [48]. 

We also demonstrate that blocking TRPM7 renders HL60-R more sensitive to the cytotoxic effects of DXR. This behavior is the opposite of what we described in LoVo. Indeed, inhibiting TRPM7 induces the acquisition of a DXR-resistant phenotype in LoVo-S [11]. These data indicate that manipulating the levels or the activity of TRPM7 interferes with the sensitivity to DXR. More studies should be devoted to this topic.

Beyond regulating cellular energy generation, mitochondria play a role in redox alterations within cancer cells [32], store Mg [12] and control cell life and death [25]. On these bases, we analyzed mitochondria in our experimental models and found no differences in the total mitochondrial content in DXR-resistant vs. sensitive cells. In HL60-R and -S also the morphology of the mitochondria remains unchanged. Significant differences emerge between LoVo-R and -S, thus confirming previous studies [10]. LoVo-S exhibit a complex mitochondrial network with lengthened mitochondria, whereas LoVo-R display round mitochondria. These organelles are very dynamic and continuously carve their shape through the fission and fusion processes. Altered mitochondrial fusion and fission underpin cancer [49,50]. In general, mitochondrial fission facilitates metastasis and drug resistance of cancer cells [49,50] and the inhibition of DRP1-mediated mitochondrial fission sensitizes ovarian cancer cells to cisplatin [51]. Therefore, we analyzed OPA1 and DRP1, markers of fusion and fission, respectively, and found that LoVo-R upregulate DRP1 and downregulate OPA1, thereby pointing to an increase of fission and a decrease of fusion. OPA1 deletion is associated with mitochondrial fragmentation and reduced cristae biogenesis [52]. Interestingly, in LoVo-R we have shown fewer, thin and disorganized cristae as well as cristolysis by electron microscopy [10]. We hypothesize that the reduced amounts of OPA1 might drive these alterations. At the moment no data are available about the regulation of OPA1 in colon cancer, but it is reported that OPA1 is markedly decreased in hepato-cellular carcinoma along with a high mitochondrial fragmentation [53]. In agreement with our findings, in colorectal cancer the activation of DRP1 leads to chemoresistance [54]. Moreover, mitochondrial fission dependent on DRP1 is essential for stemness maintenance [55] and we have previously shown that LoVo-R are more staminal than LoVo-S [13]. It will be challenging to reprogram mitochondria in LoVo cells and investigate stemness, growth rate, invasiveness and sensitivity to anticancer drugs [56]. Turning to HL-60, no differences in the amounts of OPA1 and DRP1 were detected between sensitive or resistant cells. To the best of our knowledge, no data are available about the levels of these proteins in myeloid leukemia cell lines. In agreement with our results, in murine lymphocytic leukemia L1210 cells resistant or not to cisplatin the expression of fission protein DRP1 and inner membrane fusion protein OPA1 was not significantly altered [57].

In brief, we showed that LoVo-R and HL60-R share common features that might be important to escape cytotoxicity. Indeed, both HL60-R and LoVo-R accumulate less ROS, thus being protected against oxidative damage, and more Mg, which is necessary for ATP and for antiradical defenses. These characteristics might represent a signature of DXR resistance and provide new hints to fight it. We also point to TRPM7 as a potential regulator of chemoresistance. Some questions arise: is Mg homeostasis involved in chemoresistance? Are Mg transporters playing a role? Future studies are in progress to find the answers.

## Data Availability

The data presented in this study are openly available in Dataverse at https://dataverse.unimi.it/dataverse/CIMB/ (accessed on 21 May 2021).
